# Phosphorescence lifetime microscopy and contrast-enhanced ultrasound imaging revealed strong left-to-right intraventricular shunt in anesthetized *Xenopus laevis*.

**DOI:** 10.1016/j.jphyss.2026.100098

**Published:** 2026-08-29

**Authors:** Ken Hashimoto, Momoko Ohira, Misaki Kimoto, Akiko Oda, Kosuke Tsukada, Akira Hanashima, Satoshi Mohri

**Affiliations:** a First Department of Physiology, Kawasaki Medical School, Kurashiki 701-0192, Japan; b Department of Applied Physics and Physico-Informatics, Keio University, Yokohama 223-8522, Japan

**Keywords:** Amphibian, Intracardiac shunt, Phosphorescence lifetime microscopy, Contrast-enhanced ultrasound

## Abstract

Amphibians exhibit intracardiac shunting, by which oxygen from multiple respiratory surfaces is variably distributed between systemic and pulmonary circulations. Although shunting has been extensively studied in terrestrial species that rely heavily on pulmonary respiration, much less is known about more aquatic, cutaneous-respiring species, in which cutaneously oxygenated blood returns to the right atrium, so the right–left oxygen gradient should vary with context. Moreover, the proposed role of ventricular trabeculation in limiting intraventricular mixing has not been directly demonstrated. We combined phosphorescence lifetime microscopy for in vivo pO_2_ measurement with microbubble-based contrast-enhanced ultrasound for high-resolution tracer-tracking in aquatic *Xenopus laevis*. In anesthetized animals, blood pO_2_ was uniformly low (25–40 mmHg), with little arteriovenous difference. Right-to-left shunting was minimal (8%), whereas a strong intraventricular left-to-right shunt (47%) maintained threefold greater pulmonary than systemic flow. Although its significance remains to be elucidated, these approaches provide a foundation for advancing shunt research.

## Highlights

• Intracardiac shunting in aquatic, cutaneous-respiring frog *Xenopus laevis* was studied.
• New in vivo pO_2_ microscopy and microbubble ultrasound approaches were introduced.
• Blood pO₂ was uniformly low, with little arteriovenous difference under anesthesia.
• Right-to-left shunt was minimal, but left-to-right intraventricular shunt was strong
• These approaches open new avenues for amphibian shunt research.

## 1 Introduction

The organizing principle of the mammalian cardiorespiratory system is simple: a fully separated circulation maximizes oxygen uptake by a single respiratory organ, the lungs. In amphibians, by contrast, gas exchange may involve up to four surfaces (gills, skin, lungs, and buccopharyngeal mucosa), and their relative contributions vary with species, developmental stage, and rapidly changing environmental and behavioral conditions [1]. Correspondingly, the amphibian heart—comprising variably septated atria, an undivided ventricle, and an outflow tract with an imperfect spiral valve—has been thought to permit intracardiac shunting (blood mixing), allowing flexible perfusion toward the systemic vs pulmocutaneous/pulmonary circuits [2], [3]. Explaining the functional significance of this seemingly “imperfect” design remains challenging, yet is central to understanding the evolution of circulatory separation during vertebrate terrestrialization [4], [5].

Historically, anatomical observations supported a “no-mixing” view in anurans [6]. However, extensive mid-20th-century studies using (1) oxygen analysis of blood sampled from multiple vascular sites and (2) tracer tracking after right- or left-sided cardiac injection produced highly inconsistent conclusions, ranging from no mixing to partial mixing and complete mixing [7], [8], [9], [10], [11], [12], [13], [14], [15], [16], [17], [18]. These discrepancies were attributed to species differences, habitat (terrestrial versus aquatic), the relative dependence on different respiratory surfaces, temperature, activity state, and experimental conditions, including the effects of anesthesia or pithing and whether the pericardium remained intact [18]. Subsequent studies using chronically instrumented, recovered, and conscious animals provided important clues toward resolving these issues. For example, *Rhinella marina*, a terrestrial toad that relies heavily on pulmonary respiration, exhibits a right-to-left (RL) shunt, in which systemic flow predominates over pulmocutaneous flow, at rest, but shifts to a left-to-right (LR) shunt, in which pulmocutaneous flow predominates, under conditions of increased pulmonary ventilation such as hypoxia, elevated temperature, and activity [4], [19], [20]. This reduction in pulmocutaneous vascular resistance is thought to result from both vagal withdrawal and enhanced sympathetic activity [4], [19]. However, much less is known about more aquatic, cutaneous-respiring species. In such species, oxygenated blood acquired through the skin returns to the right atrium, so the right–left atrial oxygen gradient should vary with context, and an RL shunt may even increase systemic pO_2_. Shunt dynamics under such conditions remain largely unknown. In addition, although blood entering the single ventricle from the two atria is thought to remain separated because the spongy trabeculation retains the inflow streams from each atrium [21], [22], this has not been demonstrated in vivo. To address these issues, we introduced new methods to overcome technical limitations of classical methods—especially insufficient spatiotemporal resolution and hemodynamic disturbance.

A first major limitation of prior studies is oxygenation measurement. With the exception of a few studies in conscious animals using implanted catheters [1], [23], most relied on biochemical analyses of blood sampled from multiple sites [4], [16], [17], [18], which are constrained by invasiveness, sampling-to-analysis delay, and the difficulty of obtaining multi-site samples from the same animal under identical conditions. To address this, we introduced phosphorescence lifetime microscopy (PLM) for in vivo pO_2_ measurement. PLM uses metalloporphyrin probes whose phosphorescence lifetime shortens in proportion to molecular oxygen-dependent quenching, enabling quantitative pO_2_ readout [24]. PLM allows repeated, minimally invasive pO_2_ measurements from multiple vessels and tissues without blood sampling or needle sensors. Although widely used in rodents [25], [26], [27], its application to whole-animal amphibian physiology has not been reported.

A second limitation is quantitative assessment of shunt-dependent flow partitioning. Classical tracer approaches—introducing dyes or other tracers into the right versus left side of the heart—lacked sensitivity and depth resolution, often required tracer loads large enough to alter hemodynamics, and typically required surgical exposure of the heart, further perturbing flow [7], [8], [9], [10], [11], [12], [13], [14]. We therefore adopted contrast-enhanced ultrasound (CEUS) with second-generation microbubbles. Unlike early agitated-air bubbles, modern microbubbles are small (1–5 μm filled with inert gas), stable, and reproducible, producing strong nonlinear backscatter that can be selectively detected by harmonic imaging for high-sensitivity, real-time transthoracic imaging at low doses without disturbing blood flow [28], [29]. Microbubble-based CEUS is widely used in mammals for quantifying microvascular perfusion and pathological right–left communications [30], [31], [32], but applications to physiological shunting in non-mammalian vertebrates remain rare and largely qualitative, for example using air bubbles introduced only from the right side of the heart [22], [33], [34]. Here, we applied dual-injection protocols to independently assess RL and LR shunting by injecting from the femoral vein (right side) or pulmonary vein (left side), respectively. We quantified flow partitioning from CEUS signal dynamics in outflow vessels supplying the systemic versus pulmonary circuits and also examined atrioventricular inflow, ventricular trabecular, and spiral-valved outflow regions.

By combining PLM and CEUS, we examined intracardiac shunting in the aquatic anuran *Xenopus laevis* (*X. laevis*). In this species, cutaneous oxygen uptake accounts for up to 30% of total oxygen uptake in freely behaving animals [35]. Further highlighting the importance of cutaneous respiration, embryos of *X. laevis* reared without access to air survive and mature for at least two years without breathing air [36]. We found that in anesthetized *X. laevis* under low oxygen-demand conditions, (1) blood pO_2_ was uniformly low (25–40 mmHg) with little arteriovenous difference, and (2) although RL shunting was minimal (8%), a pronounced LR shunt (47%) had already been established within the ventricles before blood encountered the spiral-valved outflow region, thereby maintaining abundant pulmonary blood flow, exceeding systemic outflow by ∼3-fold.

## 2 Materials and methods

### 2.1 Animals

Adult *X. laevis* were obtained from Hamamatsu Seibutsu Kyozai Co., Ltd. (Shizuoka, Japan) and housed in plastic tanks containing aerated, dechlorinated water at 20°C under a 12:12-h light–dark cycle. Animals were fed once weekly with commercially available sinking pellets supplemented with frozen bloodworms (Hikari Crest Cat and Vitacrin Akamushi; Kyorin Co., Ltd., Himeji, Hyogo, Japan). Male frogs weighing 55–70 g were used for experiments (36 animals in total), except for ultrasonic flowmetry, in which larger females weighing 150–180 g were used (5 animals) because of probe size constraints. Animals were acclimated at a water temperature of 20°C (for at least 2 weeks and up to 3 months), and all subsequent experiments were performed at room temperature (20–25°C). The experiments were performed during the following periods: PLM, August–September 2025; CEUS, October 2025–June 2026; and ultrasonic flowmetry, January 2026. In the PLM, CEUS, and ultrasonic flowmetry experiments described below, separate animals were used for each protocol. However, in two CEUS animals, adequate microbubble washout was confirmed shortly after the first injection from the femoral vein, and a second injection from the pulmonary vein was therefore performed sequentially. The number of animals used in each experiment is given in the figure legends. In all experiments, the pericardium was left intact to avoid nonphysiological enhancement of shunting caused by pericardial incision [18]; moreover, in CEUS, the chest wall was also left completely intact (i.e., without opening the thoracic wall). All experimental procedures were approved by the Institutional Animal Care and Use Committee of Kawasaki Medical School (Kurashiki, Okayama, Japan) and conducted in accordance with the relevant institutional, national, and international guidelines for animal welfare. All efforts were made to minimize animal suffering.

### 2.2 Anesthesia

Animals were anesthetized by immersion in 1 g/L tricaine methanesulfonate (MS-222; NACALAI TESQUE, INC., Kyoto, Japan) buffered to pH 7.0 with sodium bicarbonate for 20 min, as described previously [37]. Adequate anesthesia was confirmed by loss of the righting reflex and withdrawal reflexes. During subsequent experiments performed at room temperature (20–25°C), animals were positioned on paper towels soaked with the same anesthetic solution to keep the skin continuously moist and thereby support oxygen uptake and carbon dioxide excretion via cutaneous respiration. The time required for each experiment, including the surgical procedures, was as follows: PLM, 50 min plus an additional 10–40 min for pulmonary ventilation or cutaneous bubbling; CEUS and flowmetry, 40 min. This anesthetic condition in *X. laevis* has been reported to preserve awake-like heart rate and arterial oxygen saturation, whereas pulmonary ventilation is profoundly depressed [37], [38]. Accordingly, in our preparation, lung breathing was completely absent, whereas cutaneous gas exchange was preserved. In this anesthetized preparation, flow distribution between the pulmocutaneous and systemic circulations changed markedly in the expected directions in response to the muscarinic receptor antagonist atropine and the adrenergic agonist norepinephrine (Supplemental Fig. 1). This indicates that autonomic tone mediated by the vagal and sympathetic pathways, which are central to shunt regulation, was sufficiently preserved.

### 2.3 Phosphorescence lifetime microscopy (PLM)

Anesthetized animals were placed in the supine position and the chest was opened to expose the major vessels while leaving the pericardium intact. Only the minimum length of each target vessel required for optical measurements was carefully dissected free from surrounding tissue. Target vessels included the third aortic arch (carotid artery; 3AA), fourth aortic arch (systemic aorta; 4AA), sixth aortic arch (pulmocutaneous artery; 6AA), inferior vena cava (IVC), superior vena cava (SVC), and pulmonary vein. A phosphorescent porphyrin probe (Pd-TCPP; Pd(II) meso-tetra(4-carboxyphenyl)porphine; PdT790; Frontier Specialty Chemicals, Logan, UT, USA) was prepared at 3 mg/mL and injected into the brachial vein (50–100 μL). Immediately after injection, the animal was transferred to a custom-built perfusion bath chamber designed to immerse the dorsal half of the body in anesthetic-containing water. The bath was continuously circulated using a tubing pump (TP-20SA; AS ONE Corporation, Osaka, Japan) to maintain cutaneous gas exchange during measurements. The chamber was mounted on an upright microscope (DM LFSA; Leica Microsystems, Wetzlar, Germany) for pO_2_ measurements. The precise location of each target vessel was determined from bright-field images acquired via a CCD camera connected to the microscope side port (Digital Sight DS-Ri1; Nikon, Tokyo, Japan) and a motorized XY stage (MLS203; Thorlabs, Newton, NJ, USA). Porphyrin stock solution was dissolved in 5% bovine serum albumin in PBS for ≥ 6 h, cleared through a 0.22-μm filter, aliquoted, and stored at −30 °C until use.

After baseline pO_2_ measurements under the initial condition, pO_2_ was re-measured at the same sites in the same animals under two physiological manipulations: (1) enhanced pulmonary ventilation, and (2) enhanced cutaneous oxygen uptake. For the former, the outer cannula of an intravenous catheter (SURFLO® I.V. catheter, 20 G; Terumo, Tokyo, Japan) was used for tracheal intubation, and animals were ventilated with room air using a small animal ventilator (SN-480–7; Shinano Seisakusho Co., Ltd., Bunkyo-ku, Tokyo, Japan). Ventilation was increased to either a moderate level (10–15 breaths/min, tidal volume 0.3–0.4 mL, equivalent to 7–12% of total lung capacity for 30–40 min) or a strong level (40 breaths/min, tidal volume 1.0 mL, equivalent to 23–29% of total lung capacity for 20 min) [39]. For the latter cutaneous protocol, the circulating bath water was bubbled with a gas mixture adjusted to 40–60% O₂ at 600 mL/min for 15–20 min using a gas mixer (TK-MIGM01-O2KM; Tokken, Yokohama, Japan).

### 2.4 PLM: optical system, acquisition, and quantification

The PLM optical system was based on our previously described platform [40], with minor modifications as follows. Excitation light was provided by a frequency-doubled Q-switched Nd:YAG pulsed laser (DTL-314QT; Laser‑Export Co. Ltd.; 532 nm; pulse width <10 ns). The laser beam was directed into an upright microscope and was focused onto the measurement plane through an objective lens (HCX PL FLUOTAR 5 ×/0.15; Leica Microsystems). Phosphorescence emitted from intravascular Pd-TCPP was detected with photomultiplier tube(s) (PMT). Laser triggering and digitization of PMT signals were controlled by a custom program written in LabVIEW (National Instruments). pO_2_ was calculated from phosphorescence lifetime using the Stern–Volmer relationship as follows:

$$\frac{\tau_{0}}{\tau }\;=\;1\;+\;k_{q}\cdot \tau_{0}\cdot \left[pO_{2}\right]$$

where *k*_*q*_ is the quenching constant, and *τ*_0_ and *τ* are the phosphorescence lifetimes under anoxic conditions and in the measurement environment, respectively. Phosphorescence waveforms were sampled at 250 kHz using a 2.3-ms acquisition window per waveform. Phosphorescence signals below a predefined threshold were excluded, as these were considered regions with insufficient Pd-TCPP signal. The system was calibrated using a two-point calibration with Pd-TCPP solutions equilibrated at (i) 0% O₂ and (ii) air (20.8% O₂). The 0% O₂ condition was prepared by chemical deoxygenation with sodium sulfite (Na₂SO₃). Calibration solutions were prepared using the same Pd-TCPP formulation as for in vivo experiments. Because the injected Pd-TCPP is largely albumin-bound in the circulation and its intravascular concentration is expected to remain stable over the measurement period, the recorded phosphorescence signal was interpreted primarily as intravascular [40].

### 2.5 Contrast-enhanced ultrasound imaging (CEUS)

Anesthetized animals were placed in the supine position. Under a stereomicroscope, either a systemic vein (the femoral vein) or the pulmonary vein was cannulated as follows. The femoral vein was identified by making a small incision in one thigh and separating it from the accompanying femoral artery. The pulmonary vein was identified by making a small incision in one flank to expose the posterior region of the lung, where it was recognized as a large vein running toward the heart. For both vessels, the peripheral side was ligated, and an intravenous catheter (SURFLO® I.V. catheter, 20 G; Terumo Corporation, Tokyo, Japan) was inserted in an antegrade direction, with the cannula advanced toward the heart, thereby cannulating the vessel in an occlusive manner.

**Fig. 1:**
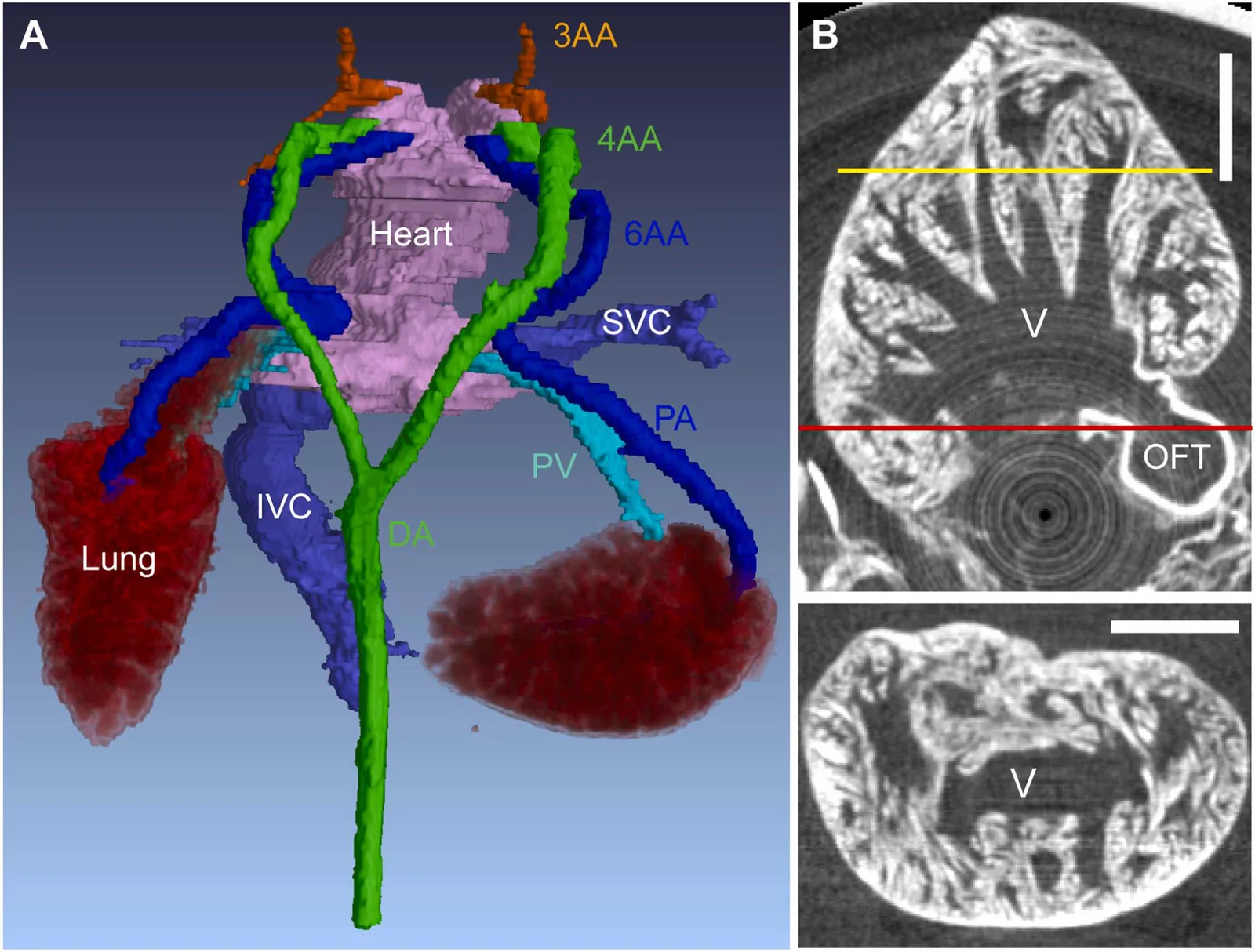
**Cardiovascular anatomy of*****X. laevis*****.** (A) Three-dimensional reconstruction of the heart and surrounding major inflow and outflow vessels obtained by contrast-enhanced CT angiography. Shown are representative images obtained from two animals. 3AA, third aortic arch artery; 4AA, fourth aortic arch artery; 6AA, sixth aortic arch artery; DA, dorsal aorta; PA, pulmonary artery; PV, pulmonary vein; IVC, inferior vena cava; SVC, superior vena cava. A rotating 3D rendering of this volume is provided in Supplemental Video 1. (B) Micro-CT visualization of ventricular internal structure obtained from a single animal. Top, longitudinal section; bottom, transverse section. V, ventricle; OFT, outflow tract. The red and yellow lines indicate the imaging planes used for contrast-enhanced ultrasound (CEUS) analysis, corresponding to the atrioventricular inflow near the cardiac base (Fig. 3D) and the ventricular trabecular region near the apex (Fig. 3E), respectively. Scale bar = 2 mm.

A high-frequency ultrasound probe (14–22 MHz) connected to an ultrasound system (Aplio 300 Platinum Series or Aplio me; Canon Medical Systems Corporation, Otawara, Tochigi, Japan) [41] was positioned to obtain one of the following cross-sections without opening the thoracic wall: (i) atrioventricular inflow near the cardiac base (red line in Fig. 1B), (ii) ventricular trabecular region near the apex (yellow line in Fig. 1B), (iii) spiral-valved outflow region, or (iv) a transverse view of the outflow tract (truncus arteriosus) encompassing one side (right or left) of the paired three aortic arch arteries (3AA, 4AA, and 6AA), as described in previous studies [14], [42]. Blood flow in the target plane was first confirmed using color Doppler (Advanced dynamic low mode). The system was then switched to contrast harmonic imaging. A second-generation microbubble contrast agent (Sonazoid®, perfluorobutane microbubbles; GE HealthCare Pharma Co., Ltd., Tokyo, Japan) was diluted in physiological saline to 1/5–1/40 of the clinical concentration, and 50 μL was injected either from the systemic venous side (femoral vein; to interrogate the cardiac right-side input) or from the pulmonary venous side (to interrogate the cardiac left-side input), followed by a small volume of physiological saline to flush the line. Microbubble dynamics within the imaging plane were recorded at 23–30 frames/s using multipulse harmonic imaging with phase or amplitude modulation.

In preliminary experiments, we identified a measurement window for outflow tract plane in which 3AA, 4AA, and 6AA ran approximately parallel to each other and were oriented as close to perpendicular to the ultrasound probe as possible; this window was then used as the reference for positioning in each animal. To minimize acoustic shadowing (i.e., attenuation of ultrasound penetration by microbubbles closest to the probe) [30], microbubble concentration was kept as low as feasible. Imaging was performed at low mechanical index (MI = 0.2), with the focal depth set slightly deeper than the target vessels. Because microbubbles typically wash out within ∼10–15 min [28] and to avoid confounding effects of residual contrast and time-dependent physiological drift, each animal generally underwent a single injection. A second injection was performed only when adequate washout was confirmed within a short interval; no differences were observed between first and second injections. For pulmonary vein injections, to account for potential ipsilateral perturbation of pulmonary venous return by cannulation, outflow tract measurements were obtained on the contralateral side. No consistent differences were observed attributable to the left–right choice of cannulation or imaging side.

### 2.6 CEUS: quantification of RL and LR shunting

Videos acquired at the outflow tract cross-section containing 3AA, 4AA, and 6AA were analyzed in ImageJ/Fiji (National Institutes of Health). For each vessel, the luminal boundary was manually traced to define a region of interest (ROI). To quantify the amount of microbubbles traversing each vessel, we computed the background-subtracted integrated intensity (Integrated Density in ImageJ) within each ROI for every frame, beginning at the first appearance of microbubbles and ending after completion of one or two cardiac cycles. The frame-wise integrated intensities were summed over this window and defined as the microbubble “flux” through the corresponding vessel. This analysis window was chosen because preliminary observations indicated that recirculating microbubbles begin to appear from approximately the third cardiac cycle onward. The analysis window of one to two cardiac cycles was defined directly from the recorded time–intensity dynamics, in which individual beats could be readily identified without electrocardiogram. Area normalization was not applied because integrated intensity is proportional to the integrated contrast signal over the entire vessel lumen and thus better reflects total passage (flux) than mean intensity. For background subtraction, the mean intensity within each ROI before microbubble arrival was used as baseline and subtracted from all subsequent measurements. When the brightest pixels approached saturation in the recorded video, brightness/contrast was adjusted prior to ROI quantification to avoid clipping.

Let *A* denote the summed microbubble flux through 3AA and 4AA, and *B* denote the flux through 6AA. We interpreted the ratio *A*:*B* as the relative distribution of injected microbubbles to the systemic versus pulmonary circuits. For injections from the systemic venous side (cardiac right-side input), RL shunt fraction was defined as:

$$\mathrm{RL}\;\mathrm{shunt}\left(\%\right)=\frac{A}{A+B}\;\times \;100$$

whereas for injections from the pulmonary venous side (cardiac left-side input), LR shunt fraction was defined as:

$$\mathrm{LR}\;\mathrm{shunt}\left(\%\right)=\;\frac{B}{A+B}\;\times \;100$$

In a completely separated circulation (as in mammals), both indices would be expected to be ∼0%. Although part of 6AA supplies cutaneous circulation, prior studies indicate that this fraction is typically small (∼20% in *Lithobates catesbeianus* [43] and ∼10–15% in *Rhinella marina* [19]. Given that our objective was to quantify the partitioning of cardiac output among the three major outflow channels, we treated the 6AA signal as representing the pulmonary outflow flux.

To validate the above quantification approach, we performed two calibration experiments using a flow phantom composed of plastic tubing with a lumen diameter comparable to the target vessels (inner diameter 1.3 mm, outer diameter 2.0 mm) connected to a syringe pump. Microbubble videos were acquired with the ultrasound probe positioned over the tubing and analyzed with the same ImageJ workflow as for in vivo data.

(1) Flow-rate series (constant microbubble concentration). Microbubble concentration was held constant (1/15 dilution relative to the clinical concentration), and syringe-pump flow rate was varied across four levels spanning the physiologically relevant range (40 mL/h [reference], 20​ mL/h [1/2], 13.3 mL/h [1/3], and 8 mL/h [1/5]). We tested whether the ImageJ-derived integrated-intensity metric scaled appropriately with imposed flow rate.

(2) Concentration series (constant flow rate). Syringe-pump flow rate was held constant (15 mL/h), and microbubble concentration was varied across four levels corresponding to the range used in vivo (1/5 dilution [reference], 1/10 [1/2], 1/15 [1/3], and 1/25 [1/5]). We tested whether the quantified flux metric scaled with microbubble concentration under fixed flow.

### 2.7 Ultrasonic flowmetry for systemic/pulmonary flows

Anesthetized animals were placed in the supine position. The chest was opened to expose the major vessels, without opening the pericardial sac (pericardium left intact). One of the paired aortic arch arteries (3AA, 4AA, and 6AA) was then carefully dissected free over the minimum length required for probe placement. In anurans, 3AA (carotid artery) is known to have substantially higher vascular resistance than 4AA and 6AA; in *Rhinella marina*, only ∼5–10% of cardiac output is reported to pass through the carotid circulation [44]. To quantify flow distribution in the present study, we used a dual-channel transit-time ultrasound flowmeter (TS420; Transonic Systems Inc., Ithaca, NY, USA). Flow was first measured in 3AA using a MA-1PRB perivascular flowprobe (Transonic Systems). The probe was then rapidly repositioned such that flow in 4AA was measured with the MA-1PRB probe while flow in 6AA was measured simultaneously with a MA-1.5PRB probe. Probe sizes were selected in preliminary experiments to match vessel diameters. Although Transonic perivascular flowprobes were factory-calibrated for absolute volume-flow measurements, the system was zeroed and checked before each experiment according to the manufacturer’s instructions.

Analog flow signals were digitized at 10 Hz using a PowerLab 8/30 data-acquisition system (ML870; ADInstruments, Dunedin, New Zealand) and analyzed in LabChart (v8.1.27; ADInstruments). For each vessel, recordings were obtained for ∼1 min under stable signal conditions, and mean flow was calculated by averaging over at least 10 consecutive cardiac cycles. No consistent differences were observed with respect to left–right vessel selection, and based on this observation and prior reports [14], [45], total cardiac output was calculated as:

Cardiacoutput=2×(F3+F4+F6)

Where *F3*, *F4*, and *F6* are the measured volume flows in 3AA, 4AA, and 6AA on one side, respectively [4]. Pulmonary-to-systemic outflow distribution was quantified as 6AA flow relative to the summed 3AA-plus-4AA flow (i.e., *F6* / (*F3* + *F4*)). Although a portion of 6AA supplies cutaneous circulation, prior studies indicate this fraction is generally small as mentioned above [19], [43]; therefore, we operationally defined the 6AA flow as pulmonary outflow. Heart rate was determined directly from the pulsatile flow waveform by counting at least 10 beats over the analyzed segment.

### 2.8 Contrast-enhanced CT analysis for cardiovascular anatomy

Anesthetized animals were placed in the supine position, and the chest was opened to expose the heart and the surrounding inflow/outflow vessels. A 2-mL contrast mixture was then injected by direct ventricular puncture, filling the ventricle and spreading broadly into the surrounding atria as well as the inflow and outflow vessels distributed throughout the trunk region. The mixture consisted of an iodinated contrast agent (Iopamiron 370; Bayer Yakuhin, Ltd.; diluted 1:14), Evans blue (0.01%, w/v), and a food-grade thickening agent (Tsururinko Quickly; Morinaga Milk Industry Clinico Co., Ltd.; 5.5%, w/v; major ingredients: dextrin, xanthan gum, calcium lactate, trisodium citrate). Evans blue was included to visually confirm the distribution of the injected solution, and the thickener was used to increase viscosity to mildly distend vessels and reduce vascular collapse during scanning. CT imaging was performed using a laboratory animal X-ray CT system (Latheta LCT-200; Hitachi Aloka Medical, Ltd.). Imaging parameters were: voxel size, 160 × 160 μm; slice thickness, 320 μm; and slice spacing, 320 μm. CT datasets were imported into VGSTUDIO MAX (v2.0; Volume Graphics GmbH) for identification of cardiac and vascular structures, segmentation, and 3D reconstruction.

### 2.9 Micro-CT analysis for intracardiac structure

Anesthetized animals were placed in the supine position. Under a stereomicroscope, IVC and pulmonary vein were cannulated, and the heart was cleared by perfusion with phosphate-buffered saline (PBS), followed by perfusion fixation with 4% paraformaldehyde (PFA). The atria and ventricle, together with short segments of adjacent vessels, were excised and post-fixed by immersion in 4% PFA for 48 h. Tissues were then rinsed in PBS, dehydrated in 70% ethanol for 1 h, and stained with 0.3% phosphotungstic acid (PTA) prepared in 70% ethanol (Nacalai Tesque; Cat. No. 27807–62) for 10 days at room temperature, adapted from previous PTA-enhanced micro-CT protocols [46], [47]. After staining, samples were rinsed in 70% ethanol and scanned on a microfocus X-ray micro-CT system (EleScan mini; Nittetsu Elex Co., Ltd.). Scans were acquired over 360° with 0.2° angular steps at 60 kV and 70 μA, with a reconstructed pixel size of 33.6 μm. Ring-artifact reduction and post-alignment corrections were applied prior to reconstruction. Reconstructed volumes were visualized in VGSTUDIO MAX (v2.1; Volume Graphics GmbH).

### 2.10 Statistical analysis

Data are presented as mean ± standard error of the mean (SEM). Comparisons between two groups were performed using two-tailed Student’s *t*-tests (paired or unpaired, as appropriate) in Microsoft Excel 2019 (Microsoft, Redmond, WA, USA). For PLM experiments involving comparisons of pO_2_ among vascular sites under each experimental condition, one-way ANOVA followed by Tukey’s post hoc multiple-comparison test was performed using IBM SPSS Statistics (v26; IBM Corp., Armonk, NY, USA). Normality was assessed in SPSS prior to parametric testing. A p value < 0.05 was considered statistically significant. Significance levels are indicated as p < 0.05 (*), p < 0.01 (**), and p < 0.001 (***). Additional statistical details, including sample sizes and exact p values, are provided in the figure legends.

## 3 Results

### 3.1 Cardiovascular anatomy of *X. laevis*

Contrast-enhanced CT angiography showed that the arterial outflow from the heart bifurcated into three paired aortic arch arteries on each side, consistent with previous descriptions [14]. The third aortic arch (3AA) continued cranially as the left and right carotid arteries, the fourth aortic arch (4AA) converged dorsally to form a single dorsal aorta supplying the trunk, and the sixth aortic arch (6AA) formed the paired pulmocutaneous/pulmonary outflow to the lungs (Fig. 1A; Supplemental Video 1). Venous return was also consistent with the expected pattern: IVC and SVC drained into the right atrium, whereas the pulmonary vein entered the left atrium. Micro-CT analysis demonstrated that the ventricle lacked an interventricular septum and contained a single central lumen surrounded by prominent spongy trabecular myocardium (Fig. 1B).

### 3.2 in vivo pO_2_ measurement using PLM shows uniformly low basal pO_2_ with little arteriovenous difference

**Fig. 2:**
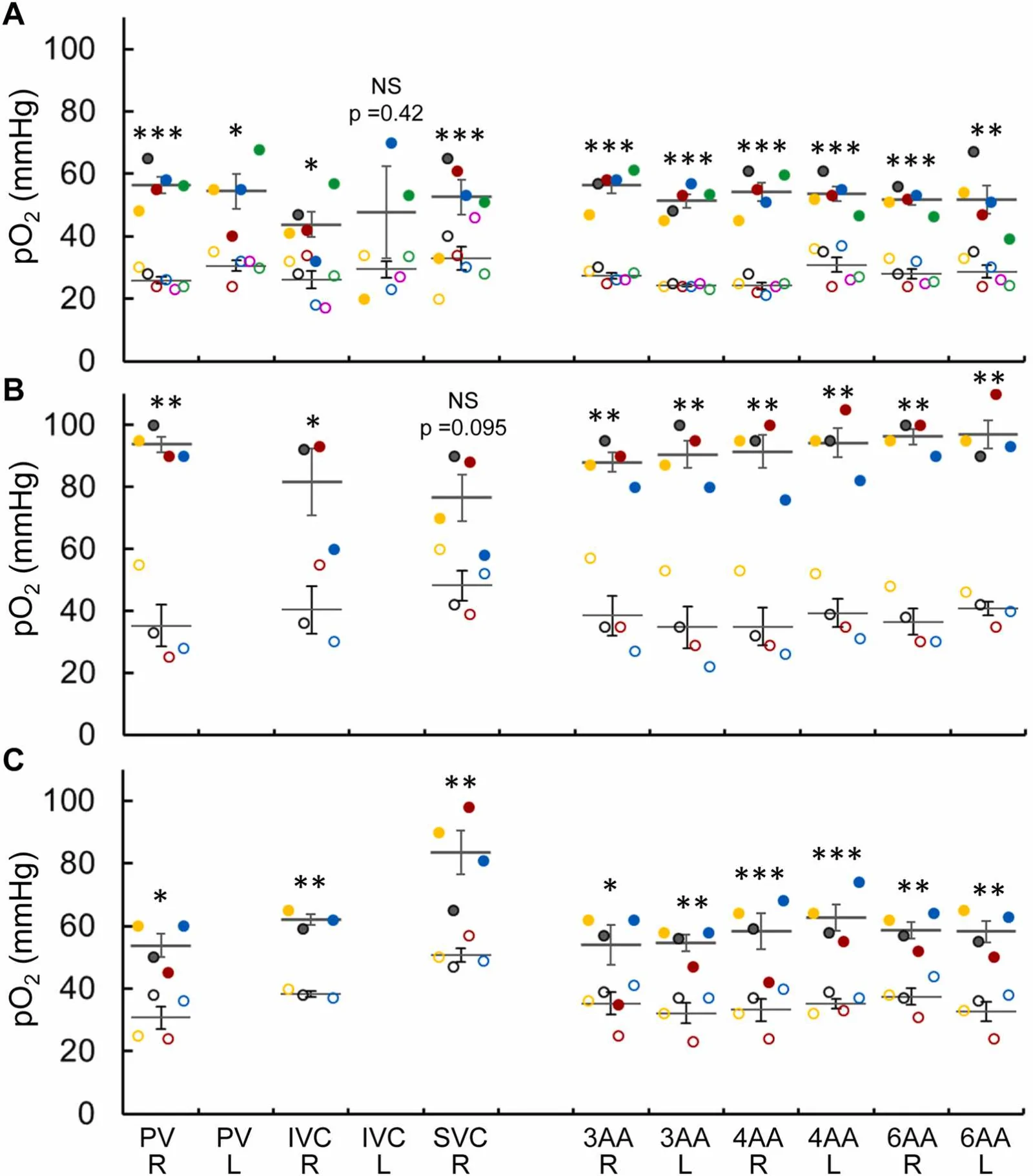
**In vivo pO**_**2**_**measurement by phosphorescence lifetime microscopy (PLM) shows uniformly low basal pO**_**2**_**with little arteriovenous differences. (A)**pO_2_ values at each vascular site under the basal (pre-manipulation) condition and during moderate lung ventilation with room air (10–15 breaths/min, tidal volume 0.3–0.4 mL, equivalent to 7–12% of total lung capacity for 30–40 min). (B) pO_2_ values under the basal condition and during strong lung ventilation with room air (40 breaths/min, tidal volume 1.0 mL, equivalent to 23–29% of total lung capacity for 20 min). (C) pO_2_ values under the basal condition and during enhanced cutaneous oxygenation by bubbling the perfusate with 40–60% O₂ (600 mL/min; 15–20 min). Protocols (A)–(C) were performed independently in separate animals, and within each protocol, measurements after manipulation were obtained in the same individual following assessment under basal conditions. Open circles indicate pre-manipulation measurements and filled circles indicate post-manipulation measurements; paired symbols with the same color in the same panel represent the same animal. Data are means ± SEM; n = 3–6 animals per group/condition. For each of the six experimental conditions (three protocols; panels A, B, and C × pre/post), pO_2_ was compared across vascular sites using one-way ANOVA followed by Tukey’s post hoc multiple-comparison test. Significant differences were detected only for comparisons involving the right superior vena cava (SVCR) in the bubbling protocol (panel C): pre-bubbling, SVCR vs PVR (p = 0.001), vs 3AAR (p = 0.021), vs 3AAL (p = 0.021), vs 4AAR (p = 0.003), vs 6AAR (p = 0.006), and vs 6AAL (p = 0.005); post-bubbling, SVCR vs PVR (p = 0.003), vs 3AAR (p = 0.003), vs 4AAR (p = 0.004), vs 4AAL (p = 0.019), vs 6AAR (p = 0.016), and vs 6AAL (p = 0.016). No other site-to-site comparisons reached significance. Within each vascular site, pre- versus post-manipulation changes were assessed using two-tailed paired Student’s *t*-tests. Post-manipulation pO_2_ increased significantly at all sites and conditions except for the two comparisons labeled “NS” in the figure. Significance levels are indicated as p < 0.05 (*), p < 0.01 (**), and p < 0.001 (***). Abbreviations: PVR/L, right/left pulmonary vein; IVCR/L, right/left inferior vena cava; SVCR, right superior vena cava; 3AAR/L, right/left third aortic arch artery; 4AAR/L, right/left fourth aortic arch artery; 6AAR/L, right/left sixth aortic arch artery.

We next measured pO_2_ in vessels entering and leaving the heart using PLM. Across all three experimental protocols (Fig. 2A, moderate lung ventilation; Fig. 2B, strong lung ventilation; Fig. 2C, enhanced cutaneous oxygenation), pO_2_ values in the basal (pre-manipulation) state were uniformly low (25–40 mmHg) at all sites. No statistically significant differences were detected among the three aortic arch arteries, and no consistent left–right differences were observed. Pulmonary arterial (6AA) and pulmonary venous pO_2_ did not differ significantly. In contrast, when systemic arteries (3AA and 4AA) were compared with systemic veins (SVC and IVC), venous pO_2_ tended to be higher, with the SVC—receiving a substantial contribution from cutaneous return—showing the highest values (statistically significant in some conditions; Fig. 2C). These basal patterns may be consistent with the physiological state of this preparation: anesthesia reduces oxygen demand, pulmonary ventilation is absent, yet cutaneous gas exchange is maintained in the perfusion bath. When air ventilation was imposed to promote lung breathing, pO_2_ increased across measured sites in a ventilation-intensity–dependent manner, and the rise tended to be larger in the pulmonary vein than in the systemic veins (Fig. 2A, B). Conversely, when cutaneous oxygen uptake was enhanced by bubbling the perfusate, pO_2_ increased across sites, with a larger rise tending to occur in systemic venous blood than in pulmonary venous blood (Fig. 2C). Together, these results support the validity of our PLM-based pO_2_ measurements and the effectiveness of our lung- versus skin-ventilation manipulations.

### 3.3 CEUS quantifies strong LR shunting and localizes a major site of mixing to the ventricle

**Fig. 3:**
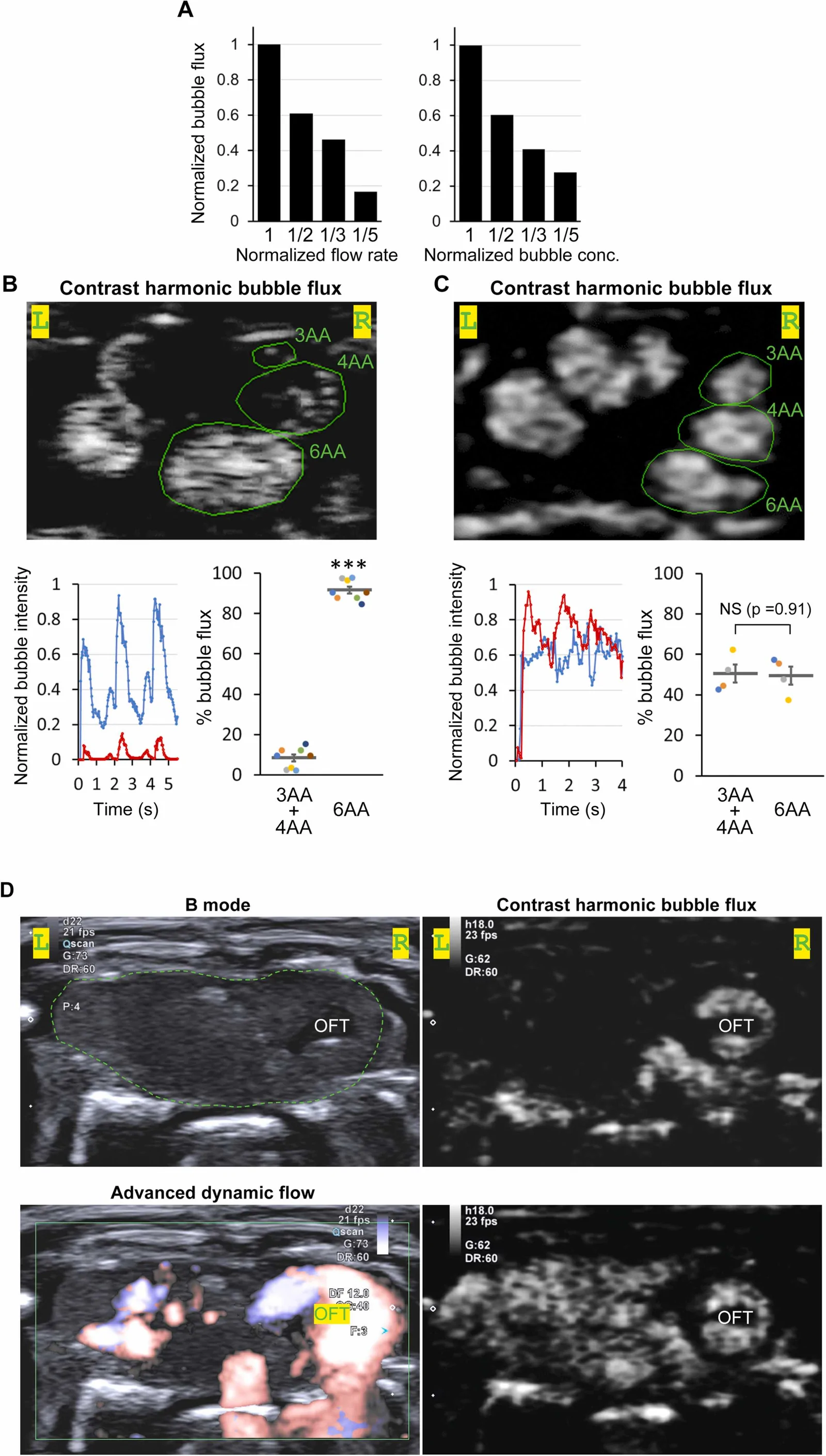

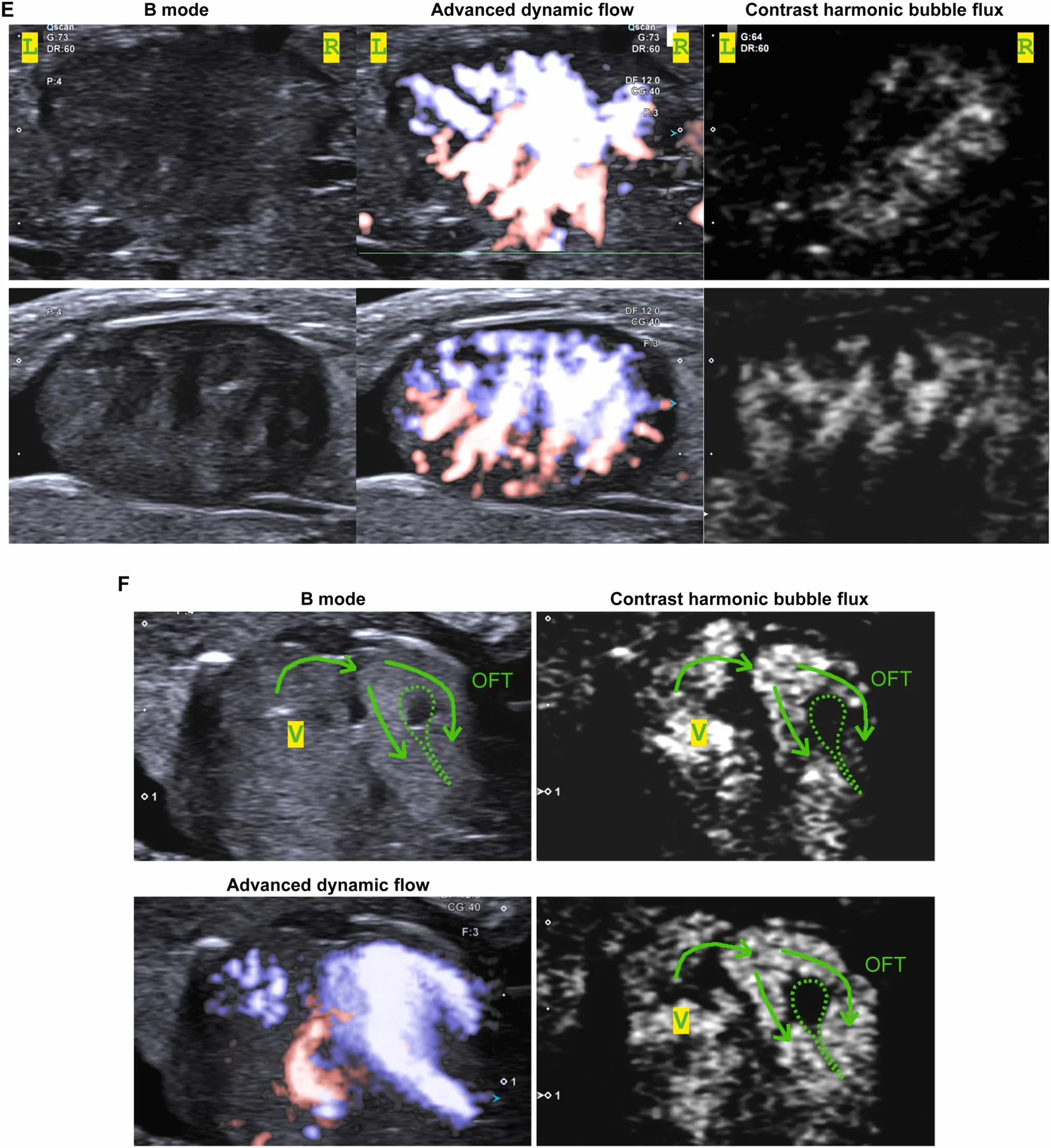
**Contrast-enhanced ultrasound (CEUS) quantifies strong LR shunting and localizes a major site of mixing to the ventricle.** (A) Flow-phantom calibration using plastic tubing comparable in size to the target outflow vessels (inner diameter 1.3 mm; outer diameter 2.0 mm). Left: Microbubble concentration was held constant and syringe-pump flow rate was varied across four levels (40 mL/h [reference], 20 mL/h [1/2], 13.3 mL/h [1/3], and 8 mL/h [1/5]). Quantified microbubble “flux” values are normalized to the reference flow (40 mL/h = 1). Right: Flow rate was held constant (15 mL/h) and microbubble concentration was varied across four levels (1/5 dilution [reference], 1/10 [1/2], 1/15 [1/3], and 1/25 [1/5]). Flux values are normalized to the reference concentration (1/5 dilution = 1). Conc. = concentration. (B–C) Quantification of right-to-left (RL) shunting after microbubble injection from the systemic venous side (femoral vein; cardiac right-side input) (B) and left-to-right (LR) shunting after microbubble injection from the pulmonary venous side (cardiac left-side input) (C). Top: Representative contrast harmonic images showing microbubble passage through the outflow tract vessels (one side, right or left, of the paired three arteries; third aortic arch artery: 3AA, fourth aortic arch artery: 4AA, and sixth aortic arch artery: 6AA). Green outlines indicate the manually defined ROIs for each vessel. 3AA appears smaller than 4AA and 6AA, consistent with its higher vascular resistance [44]; particularly evident in B). L and R indicate image orientation. Full videos are provided in Supplemental Video 2 and 3. Bottom left: Representative time–intensity traces immediately after contrast arrival. Red, summed signal from 3AA plus 4AA; blue, signal from 6AA. Traces are normalized such that peak intensity approaches 1. Bottom right: Quantified RL shunt (B) or LR shunt (C). n = 8 animals per group (B) and n = 6 animals per group (C). Symbols with the same color in the same panel denote measurements from the same animal. ***P < 0.001 or not significant (NS) versus the combined 3AA + 4AA signal (paired two-tailed Student’s *t*-test). Data are means ± SEM. (D) Representative contrast harmonic microbubble-flux images at the atrioventricular inflow plane near the cardiac base during microbubble injection from the systemic venous side (top right) or pulmonary venous side (bottom right). For the same imaging plane, a pre-injection B-mode image (top left) and color Doppler image acquired with advanced dynamic flow mode (bottom left) are shown. L and R indicate image orientation. Green dashed lines indicate the approximate epicardial contour inferred from the B-mode image shown for visual clarity. OFT, outflow tract. In this animal, a second injection (pulmonary venous) was performed after confirming adequate washout following the systemic venous injection. Full videos are provided in Supplemental Videos 4–6. Shown are representative images obtained from four animals (the systemic venous injection) and three animals (the pulmonary venous injection). (E) Representative echocardiographic images at the ventricular trabecular plane near the apex during microbubble injection from the systemic venous side (top row) or pulmonary venous side (bottom row). For each injection, the corresponding pre-injection B-mode image (left), advanced dynamic flow color Doppler image (middle), and contrast harmonic microbubble-flux image (right) are shown. L and R indicate image orientation. Full videos are provided in Supplemental Videos 7–10. Shown are representative images obtained from three animals (the systemic venous injection) and two animals (the pulmonary venous injection). (F) Representative contrast harmonic microbubble-flux images at the spiral-valved outflow region using a long-axis approach from the right lateral chest wall during microbubble injection from the systemic venous side (top right) or pulmonary venous side (bottom right). For the same imaging plane, a pre-injection B-mode image (top left) and color Doppler image acquired with advanced dynamic flow mode (bottom left) are shown. The region enclosed by the dashed line and the two arrows running along both sides indicate the two blood-flow channels within the outflow tract (OFT) separated by the spiral valve. Another arrow indicates blood flow from the outlet on the anatomical right side of the ventricular (V) base, which appears on the superficial side in this right lateral chest-wall approach, into the outflow tract. In this animal, a second injection (pulmonary venous) was performed after confirming adequate washout following the systemic venous injection. Full videos are provided in Supplemental Videos 11–13. Shown are representative images obtained from four animals each for systemic and pulmonary venous injections.

The PLM results above suggested that blood entering the right and left sides of the heart may undergo appreciable mixing via intracardiac shunting. We therefore performed CEUS-based shunt analysis. In flow-phantom calibration experiments using plastic tubing comparable in size to the target vessels, the ImageJ-derived microbubble “flux” metric varied in accordance with both syringe-pump flow rate (Fig. 3A, left) and microbubble concentration (Fig. 3A, right). These results support the overall validity of our quantitative CEUS workflow for estimating relative contrast passage through the outflow vessels. Following microbubble injection from the systemic venous side (cardiac right-side input), the majority (92%) of contrast traversed 6AA (pulmonary outflow), whereas a smaller fraction entered the systemic outflow through 3AA/4AA (RL shunt = 8%; Fig. 3B; Supplemental Video 2). In contrast, after injection from the pulmonary venous side (cardiac left-side input), only 53% of contrast entered the systemic outflow and a substantial fraction (47%) recirculated into the pulmonary outflow, indicating a pronounced left-to-right shunt (LR shunt = 47%; Fig. 3C; Supplemental Video 3).

To localize where shunting arises within the heart, we next performed CEUS observations at three additional planes: the atrioventricular inflow near the cardiac base (Fig. 3D; Supplemental Videos 4–6), the ventricular trabecular near the apex (Fig. 3E; Supplemental Videos 7–10), and the spiral-valved outflow region (Fig. 3F; Supplemental Videos 11–14). With systemic venous injection, microbubbles entering from the right atrium preferentially filled the right side of the ventricle at the inflow level (Fig. 3D, top right; Supplemental Videos 4 and 6), and a degree of right-side preference remained evident within the trabecular region, with limited spread to the left trabeculae (Fig. 3E, top right; Supplemental Videos 7 and 8). This preferential flow pattern was maintained in the spiral-valved outflow region, where the microbubbles selectively passed through only one of the two channels partitioned by the spiral valve (the image-left, ventricle-proximal channel among the two channels running from top to bottom shown in Fig. 3F, top right; Supplemental Videos 11, 13, and 14). In contrast, with pulmonary venous injection, microbubbles entering from the left atrium rapidly dispersed throughout both sides of the ventricle at the inflow plane (Fig. 3D, bottom right; Supplemental Videos 5 and 6), with a similar bilateral distribution in the trabecular region (Fig. 3E, bottom right; Supplemental Videos 9 and 10). This bilateral flow pattern was also preserved in the spiral-valved outflow region, where the microbubbles distributed evenly into both channels separated by the spiral valve (Fig. 3F, bottom right; Supplemental Videos 12, 13, and 14). Together, these observations indicate that the pronounced LR shunt is already established within the ventricle, and is maintained as blood passes through the spiral-valved outflow region. Such LR shunting is expected to increase pulmonary blood flow by returning a substantial fraction of left-heart inflow back to the pulmonary circuit.

### 3.4 Ultrasonic flowmetry reveals abundant pulmonary blood flow

**Fig. 4:**
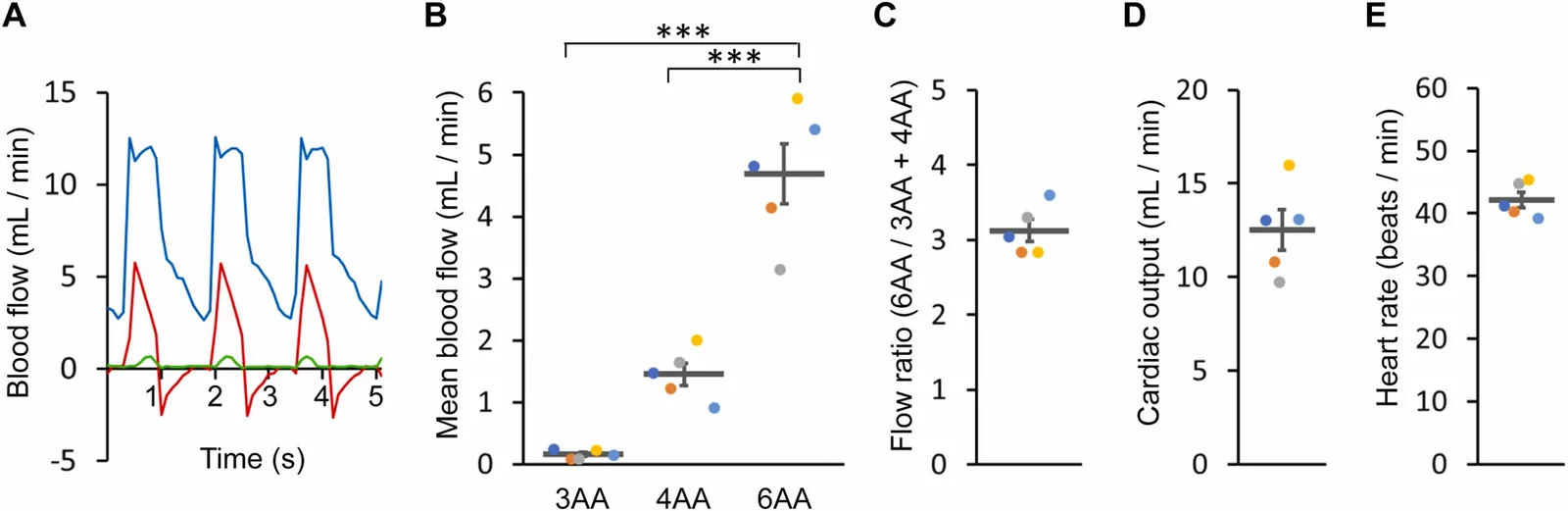
**Ultrasonic flowmetry reveals abundant pulmonary blood flow.** (A) Representative transit-time ultrasonic flow waveforms recorded from the third (green), fourth (red), and sixth (blue) aortic arch arteries. (B) Quantification of mean volume flow in each vessel. ***P < 0.001 versus 3AA or 4AA (two-tailed unpaired Student’s *t*-test). 3AA, third aortic arch artery; 4AA, fourth aortic arch artery; 6AA, sixth aortic arch artery. (C) Pulmonary-to-systemic outflow ratio, calculated by dividing the mean flow in 6AA by the combined mean flow in 3AA plus 4AA. (D) Cardiac output, estimated as twice the sum of the mean flows measured in 3AA, 4AA, and 6AA (measured on one side). (E) Heart rate, counted directly from the pulsatile flow trace over a segment containing at least 10 beats. For panels B–E, n = 5 animals per group. Data are mean ± SEM.

We next quantified volume flow in the three aortic arch arteries leaving the heart using transit-time ultrasound flowmetry (Fig. 4A). Consistent with prior observations in *Rhinella marina*, flow in 3AA (carotid) was markedly smaller than in the other two arches [44]. Representative flow waveforms further revealed distinct hemodynamic profiles between 4AA and 6AA: the former exhibited a brief period of retrograde flow during diastole, whereas 6AA showed a higher systolic peak and a more sustained high-flow phase, with persistent antegrade flow maintained throughout diastole. Quantitatively, mean flow in 6AA (pulmonary outflow) was approximately 3.1-fold greater than the summed mean flow in 3AA plus 4AA (systemic outflow; Fig. 4B, C). This marked predominance of the 6AA flow is consistent with substantial left-to-right recirculation into the pulmonary circuit as indicated by the CEUS analysis (Fig. 3C). The mean cardiac output was 12.5 mL/min (Fig. 4D), and heart rate was 42.2 beats/min (Fig. 4E).

## 4 Discussion

In this study, we introduce a new direction for the 150-year history of intracardiac shunt research in anurans by applying new approaches (PLM and CEUS) to aquatic *X. laevis* for the first time, thereby addressing key technical limitations of earlier work, including limited detection resolution and hemodynamic perturbations. Under anesthetized, low-oxygen-demand conditions, in which pulmonary breathing was abolished whereas cutaneous respiration was maintained, we found: (1) intravascular pO_2_ was uniformly low (25–40 mmHg) across sites, with little arteriovenous difference (Fig. 2); (2) RL shunting was small (8%; Fig. 3B), whereas a pronounced LR shunt (47%; Fig. 3C) had already been established within the ventricles before blood encountered the spiral-valved outflow region, thereby maintaining abundant pulmonary blood flow, exceeding systemic outflow by ∼3-fold (Fig. 4A–C).

Only one previous study [14] reported a similar shunt pattern in anesthetized *X. laevis*—namely, small RL shunting accompanied by large LR shunting—but neither oxygenation nor blood flow was measured, and the physiological significance was not discussed in depth. This shunt pattern contrasts with the predominant RL shunt at rest observed in conscious terrestrial *Rhinella marina* [4], [19], [20]. The major differences between those studies and the present study are (1) experimental conditions (conscious vs. anesthetized) and (2) differences in species/habitat (terrestrial vs. aquatic). One potential concern is that MS-222 may have induced a nonphysiological increase in pulmocutaneous blood flow by suppressing vagal tone to the pulmocutaneous vasculature. However, the strong responsiveness to atropine and norepinephrine indicates that autonomic tone mediated by the vagal and sympathetic pathways was sufficiently preserved in our preparation (Supplemental Fig. 1). Previous studies have likewise demonstrated responsiveness to acetylcholine and atropine in MS-222-anesthetized *X. laevis* [45] and *Rhinella marina* [48]. Therefore, while our anesthetized preparation cannot be considered physiologically equivalent to conscious animals, we consider that it still allows interpretation of shunt dynamics under the specific condition, i.e., predominant cutaneous respiration without pulmonary ventilation. Accordingly, the discrepancy in findings may instead reflect differences in habitat use (terrestrial vs. aquatic). In *Rhinella marina*, a terrestrial species thought to depend strongly on pulmonary respiration, an RL shunt would be expected to lower systemic oxygenation by mixing oxygen-poor blood from the right side of the heart. In contrast, in our preparation of aquatic *X. laevis*, pulmonary breathing was abolished whereas cutaneous respiration was maintained; under these conditions, an RL shunt could instead increase systemic oxygenation. This led us to speculate that, in this state, reduced RL shunting diverts cutaneously acquired oxygen into the pulmocutaneous circuit, where it is retained by LR shunting, thereby keeping systemic oxygenation low. In other words, the apparently discrepant findings may be explained if both represent a common response aimed at preventing an unnecessary rise in systemic oxygenation at rest. The uniformly low pO_2_ values (25–40 mmHg) (Fig. 2) may thus reflect an adaptive physiological strategy, rather than merely a passive consequence of limited oxygen transport. This further implies that, when right side-derived oxygen uptake exceeds a certain level, the positive correlation between pulmonary ventilation and pulmonary blood flow [49] may not apply.

In a broader developmental and evolutionary context, the present *X. laevis* preparation may resemble the mammalian fetus in several respects: both exist in an aquatic environment, lack pulmonary ventilation, receive oxygenated blood primarily through the right-sided circulation, and maintain blood pO_2_ at similarly low levels (25–40 mmHg) [50]. In the mammalian fetus, oxygenated blood entering the right-sided circulation from the placenta is preferentially directed to the systemic circulation through specialized shunt pathways, including the foramen ovale and ductus arteriosus. This strategy is generally interpreted as a mechanism for maximizing systemic oxygenation. However, this idea has not been directly demonstrated, and given that fetal blood pO_2_ is maintained at a low level and fetal oxygen demand is relatively modest, its actual physiological significance remains uncertain. In our *X. laevis* preparation, oxygenated blood entering the right-sided circulation from the skin could be retained within the pulmocutaneous circulation by minimizing RL shunting and maintaining a large LR shunt, together with abundant pulmocutaneous blood flow, leading us to consider that this may help prevent an unnecessary increase in systemic oxygenation. These considerations led us to speculate that the mammalian fetus and our *X. laevis* may use different strategies to achieve a common goal: maintaining an adaptive low-oxygen physiological state.

An important question is what advantages may be conferred by maintaining a low-oxygen state in these organisms. Among several possible explanations, previous evidence linking low-oxygen environments to cardiac regeneration may provide one possible clue. For example, amphibians living in low-oxygen environments, such as axolotls, retain robust cardiac regenerative capacity. Similarly, fetal mice exhibit cardiac regenerative capacity in the low-oxygen intrauterine environment, whereas this capacity is lost after birth in the higher-oxygen postnatal environment [51], [52] and can be reacquired when adult mice are exposed to hypoxic conditions [53]. Puente et al. [51] showed that, during the first postnatal week, reactive oxygen species (ROS), oxidative DNA damage, and DNA damage response (DDR) markers significantly increase in the heart, and that postnatal hypoxemia, ROS scavenging, or inhibition of DDR all prolong the postnatal proliferative window of cardiomyocytes, whereas hyperoxemia and ROS generators shorten it. These findings provide strong evidence that minimizing oxidative stress through the maintenance of a low-oxygen environment is critical for preserving cardiac regenerative capacity, an important physiological trait. Although oxidative stress and cardiac regeneration were not directly assessed in the present study, these previous findings support the speculation that one possible significance of maintaining a low-oxygen environment may be, at least in part, to limit oxidative stress. If this is the case, the role of the circulatory system, including intracardiac shunting, may not simply be to maximize systemic oxygen delivery.

This hypothesis must be tested in chronically instrumented, conscious *X. laevis*, except for CEUS, which can only be performed under anesthesia. Experiments under a variety of conditions—including surfacing versus submergence, manipulations of temperature and ambient O_2_/CO_2_, and stimulation of oxygen consumption with uncoupling agents such as dinitrophenol—should provide important insights. Furthermore, even in anesthetized animals, it would be worthwhile to test whether an RL shunt similar to that observed in *Rhinella marina* emerges when the lungs are ventilated while the bathing water is rendered hypoxic.

Alternatively, the strong LR shunt may serve to facilitate CO_2_ elimination through the lungs. In vertebrates, the ventilatory process is the rate-limiting step for CO_2_ excretion, and LR shunting, by providing greater blood flow to the pulmonary vasculature, may therefore contribute to CO_2_ elimination during periods of high metabolic demand [54], [55]. However, in amphibians, CO_2_ excretion generally occurs predominantly across the skin rather than the lungs [45]. Given that more than 85% of CO_2_ is eliminated through the skin in freely behaving *X. laevis* [35], the validity of this hypothesis remains to be tested.

A prevailing view has proposed that spongy ventricular trabeculation prevents blood mixing by retaining inflow streams from each atrium [21], [22]. Our CEUS observations at the trabecular plane clearly contradict this, at least for left-atrial inflow (Fig. 3E). Combined with the atrioventricular inflow images (Fig. 3D) and the spiral-valved outflow images (Fig. 3F), the data suggest the following mechanism in *X. laevis*: right-atrial inflow preferentially occupies the right side of the ventricle with limited spread to the left trabeculae and reaches the outflow tract largely segregated, whereas left-atrial inflow rapidly spreads across the whole ventricle, partially mixes with right-atrial blood, and reaches the outflow tract—thereby generating a dominant intraventricular LR shunt before blood encounters the spiral-valved outflow region. Micro-CT further revealed a discernible central lumen surrounded by trabeculae (Fig. 1B), which may provide an additional compartment for mixing. The mechanism by which right- versus left-sided inflow exhibits differential mixing behavior remains unknown. It is natural to assume that fluid entering a single undivided chamber will spread throughout the chamber according to the magnitude of its flow and pressure. Accordingly, a simple hypothesis can be proposed in which the relative magnitude of right versus left input determines the pattern of intraventricular mixing: when left inflow exceeds right inflow, the left inflow spreads toward the right side of the ventricle, as observed in the present study, thereby generating an LR shunt; when right inflow exceeds left inflow, the reverse occurs (RL shunt); and when the two are equal, no shunt occurs. The difference between right and left inflow is determined by the difference between systemic and pulmocutaneous arterial outflow, and the factor most likely to influence this difference is pulmocutaneous vascular resistance, which previous studies have shown to be subject to large, state-dependent changes [4], [56]. These changes would alter the flow balance between both circulations, thereby allowing fine adjustment of intracardiac mixing.

In summary, we found a prominent intraventricular LR shunt accompanied by abundant pulmonary blood flow in anesthetized *X. laevis*, which relied on cutaneous but not pulmonary respiration. Amphibians are a highly heterogeneous group, and these findings therefore cannot be extrapolated to other species. It should also be emphasized that such an LR shunt is unlikely to occur in terrestrial species that depend more strongly on pulmonary respiration. These findings were made possible by the first application of new approaches to anuran shunt research. PLM enabled multi-site, within-animal pO_2_ measurements without blood sampling and hemodynamic disturbance. Microbubble-based CEUS quantified flow partitioning, achieving minimally invasive, transthoracic tracer tracking with high spatiotemporal resolution. This capability enabled independent and quantitative assessment of RL versus LR shunting, which has been difficult with classical methods. Although the physiological significance remains to be fully elucidated, applying these tools across various environmental and behavioral settings offers a promising route to clarify the functional logic of amphibian cardiorespiratory control.

### 4.1 Limitations

Several limitations should be considered when interpreting our findings. First, the possible effects of MS-222 anesthesia cannot be excluded. As discussed above, autonomic tone appeared to be sufficiently preserved in our preparation under anesthesia. Nevertheless, MS-222 has been reported to induce sympathetically mediated tachycardia in *Rhinella marina* [57], which could potentially enhance intracardiac shunting through tachycardia-induced flow turbulence [4], [58]. This effect, however, was not evident in the present anesthetized *X. laevis*, in which heart rate was 42.2 beats/min (Fig. 4E), comparable to values reported for normal awake animals at similar temperatures (18–21°C), namely 40–50 beats/min [37], [38]. Moreover, the anesthetic protocol used in the present study, 1 g/L MS-222 for 20 min, has been shown to maintain heart rate and arterial oxygen saturation at near-awake levels [37], [38], [59]. However, residual effects of anesthesia relative to the conscious state cannot be ruled out and the physiological significance of the prominent LR shunt observed in the present study should be interpreted with caution. Second, CEUS-based shunt quantification depends on consistent identification of the three target outflow vessels (3 AA, 4AA, and 6AA). Based on pilot experiments, we selected an imaging window where these vessels run approximately parallel to one another and are oriented nearly perpendicular to the ultrasound probe. Nevertheless, small inter-individual differences in vessel geometry and probe positioning could introduce minor misregistration across animals. In addition, CEUS is susceptible to acoustic shadowing as mentioned above. Although we minimized microbubble concentration and optimized focal depth and acoustic settings to reduce this artifact, some residual shadowing may have led to underestimation of signal intensity in deeper regions. Third, the phantom calibration experiments supported the general validity of the ImageJ-derived “flux” metric, but the relationships were not perfectly linear (Fig. 3A). This nonlinearity likely reflects practical limits of CEUS, including finite spatial/temporal resolution and depth-dependent nonlinearities introduced by residual shadowing. Such effects are difficult to eliminate completely with current ultrasound-based approaches. Accordingly, we view our shunt quantification as an operationally robust and consistent metric. Fourth, pO_2_ measurements alone, without concomitant measurements of oxygen saturation or hemoglobin concentration, did not allow us to assess true oxygen content or systemic oxygen delivery. Integrating PLM-based PO₂ measurements with complementary measurements of these parameters would strengthen future studies. Fifth, the potential effects of thoracotomy should be considered. In the present study, which targeted vessels around the heart, CEUS could be performed under closed-chest conditions, whereas PLM and flowmetry had to be performed under open-chest conditions because they required direct vascular access. In mammals, respiration depends on negative intrapleural pressure, and loss of this negative pressure after thoracotomy can cause serious respiratory and hemodynamic disturbances, such as lung collapse, ventilation/perfusion mismatch, and reductions in venous return and cardiac output. In contrast, amphibians ventilate their lungs by positive-pressure buccal pumping and do not depend on negative intrapleural pressure as in mammals [60]. Therefore, such thoracotomy-related disturbances are unlikely to occur. Consistent with this, in a preliminary PLM analysis, the pO_2_ values measured under closed-chest conditions in the femoral artery and femoral vein, which were accessible without thoracotomy, were generally consistent with the values measured under open-chest conditions in the corresponding central vessels, namely the fourth aortic arch/systemic aorta (4AA) and the inferior vena cava (IVC), respectively. Thus, we consider it unlikely that thoracotomy critically affected cardiorespiratory physiology under the present conditions. Nevertheless, the potential effects of the open-chest preparation cannot be completely excluded, and the results should be interpreted with appropriate caution. Sixth, male animals were used for most experiments, whereas female animals were used for flowmetry because of probe size constraints. Therefore, we cannot exclude the possibility that sex differences may have influenced shunt dynamics. Seventh and finally, temperature, season, and hibernation- or aestivation-like metabolic states can markedly influence amphibian cardiorespiratory physiology. In the present study, animals were acclimated at 20°C, and all subsequent experiments were performed at room temperature (20–25°C). The experiments were performed during the following periods: PLM, August–September 2025; CEUS, October 2025–June 2026; and flowmetry, January 2026. Thus, we cannot exclude the possibility that the maximum 5°C difference in temperature and seasonal changes may have influenced shunt dynamics. Furthermore, *X. laevis* is known to undergo aestivation during the dry season [61]. Accordingly, future studies examining shunt dynamics under different temperature ranges, seasons, and aestivation-like metabolic states that reflect the natural ecology of this species would be valuable.

## CRediT authorship contribution statement

**Misaki Kimoto:** Methodology, Investigation. **Momoko Ohira:** Writing – review & editing, Methodology, Investigation. **Ken Hashimoto:** Writing – review & editing, Writing – original draft, Validation, Project administration, Methodology, Investigation, Funding acquisition, Formal analysis, Conceptualization. **Satoshi Mohri:** Writing – review & editing, Project administration, Funding acquisition, Conceptualization. **Akira Hanashima:** Writing – review & editing, Supervision, Funding acquisition, Data curation. **Kosuke Tsukada:** Writing – review & editing, Resources, Methodology, Funding acquisition. **Akiko Oda:** Formal analysis, Data curation.

## Ethics declaration

This study was conducted in accordance with the ARRIVE (Animal Research: Reporting of In Vivo Experiments) guidelines. Ethics committee approval was not required. This study used only animal subjects and did not involve human participants. Thus, human ethics committee approval was not required. The study was reviewed and approved by the Institutional Animal Care and Use Committee.

## Declaration of generative AI and AI-assisted technologies in the manuscript preparation process

During the preparation of this work the authors used ChatGPT (GPT-5) by OpenAI in order to improve the readability of the manuscript. After using this tool, the authors reviewed and edited the content as needed and take full responsibility for the content of the article.

## Funding

This work was supported by JSPS KAKENHI Grant Number 24K22413, 24K03270, 24K03254, 23H00556 to K.H., A.H., and S.M. from the Ministry of Education, Culture, Sports, Science, and Technology of Japan, and was also supported by Research Project Grants from 10.13039/501100009158Kawasaki Medical School.

## Declaration of Competing Interest

The authors declare that they have no known competing financial interests or personal relationships that could have appeared to influence the work reported in this paper.

## Data Availability

All data supporting the findings of this study are available from the corresponding author upon request.
