# Supplementary material for: Phosphorescence lifetime microscopy and contrast-enhanced ultrasound imaging revealed strong left-to-right intraventricular shunt in anesthetized Xenopus laevis
Source: J Physiol Sci. 2026 Aug 29;76(3):100098. doi: 10.1016/j.jphyss.2026.100098 (PMC13570341; doi:10.1016/j.jphyss.2026.100098)
Supplement: Supplementary file 1 [file mmc2.pdf]

Supple Fig.1

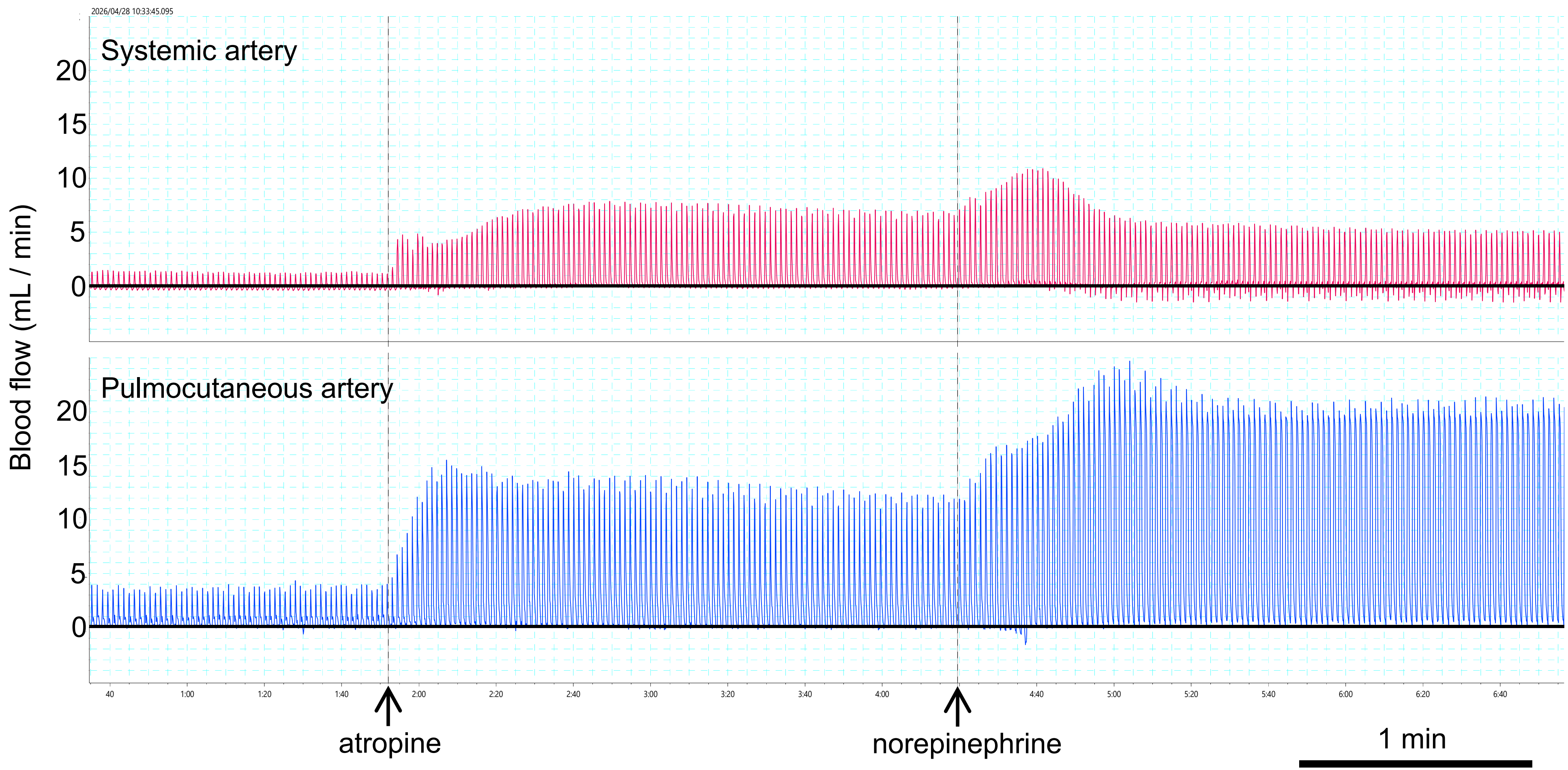

Supplemental Figure 1. Autonomic tone mediated by the vagal and sympathetic pathways, which are central to shunt regulation, remained sufficiently preserved in our anesthetized *X. laevis*.

Representative transit-time ultrasonic flow waveforms recorded from the pulmocutaneous artery (sixth aortic arch, blue) and the systemic artery (fourth aortic arch, red) in anesthetized *X. laevis*. In this example, the basal flow ratio between the two arteries was approximately 4:1. Intravenous administration of atropine (2 mg/kg, arrow) promptly increased pulmocutaneous flow to ~4.8 times the basal level, and subsequent administration of norepinephrine (200 μg/kg, arrow) increased it further to ~9.4 times the basal level. Bar, 1 min. For details of ultrasonic flowmetry, see Materials and Methods.
