# Supplementary material for: Phosphorescence lifetime microscopy and contrast-enhanced ultrasound imaging revealed strong left-to-right intraventricular shunt in anesthetized Xenopus laevis
Source: J Physiol Sci. 2026 Aug 29;76(3):100098. doi: 10.1016/j.jphyss.2026.100098 (PMC13570341; doi:10.1016/j.jphyss.2026.100098)
Supplement: Supplementary file 2 [file mmc1.docx]

**Supplemental Materials**

**Supplemental Video 1. Three-dimensional reconstruction of the heart and surrounding major inflow and outflow vessels obtained by contrast-enhanced CT angiography.**

3AA, third aortic arch artery; 4AA, fourth aortic arch artery; 6AA, sixth aortic arch artery; DA, dorsal aorta; PA, pulmonary artery; PV, pulmonary vein; IVC, inferior vena cava; SVC, superior vena cava.

**Supplemental Video 2,3. Representative CEUS imaging at the outflow tract vessels.**

Contrast harmonic microbubble-flux images during microbubble injection from the systemic venous side (femoral vein; cardiac right-side input, Video 2) or pulmonary venous side (cardiac left-side input, Video 3). Green outlines indicate the manually defined ROIs for each target vessel (one side, right or left, of the paired three aortic arch arteries). L and R indicate image orientation. In the systemic venous injection (Video 2), it was clearly evident that once more than three cardiac cycles had elapsed after microbubble appearance, recirculating microbubbles became superimposed on the signal. 3AA, third aortic arch artery; 4AA, fourth aortic arch artery; 6AA, sixth aortic arch artery.

**Supplemental Video 4–6. Representative CEUS imaging at the atrioventricular inflow plane near the cardiac base.**

Contrast harmonic microbubble-flux images during microbubble injection from the systemic venous side (femoral vein; cardiac right-side input, Video 4) or pulmonary venous side (cardiac left-side input, Video 5). For the same imaging plane, a pre-injection B-mode image (left part of Video 6) and color Doppler image acquired with advanced dynamic flow mode (right part of Video 6) are provided. L and R indicate image orientation. OFT, outflow tract. In this animal, a second injection (pulmonary venous) was performed after confirming adequate washout following the systemic venous injection. In the systemic venous injection (Video 4), it was clearly evident that once more than three cardiac cycles had elapsed after microbubble appearance, recirculating microbubbles became superimposed on the signal.

**Supplemental Video 7–10. Representative CEUS imaging at the ventricular trabecular plane near the cardiac apex.**

Contrast harmonic microbubble-flux images during microbubble injection from the systemic venous side (femoral vein; cardiac right-side input, Video 7) or pulmonary venous side (cardiac left-side input, Video 9). For each injection, the corresponding pre-injection B-mode image (left part of Video 8 for systemic injection and left part of Video 10 for pulmonary injection) and advanced dynamic flow color Doppler image (right part of Video 8 for systemic injection and right part of Video 10 for pulmonary injection) are provided. L and R indicate image orientation. In the systemic venous injection (Video 7), microbubbles entering from the right atrium preferentially filled the right side of the ventricle, although some spread toward the left side became apparent.

**Supplemental Video 11–13. Representative CEUS imaging at the spiral-valved outflow region** **using a long-axis approach from the right lateral chest wall.**

Contrast harmonic microbubble-flux images during microbubble injection from the systemic venous side (femoral vein; cardiac right-side input, Video 11) or pulmonary venous side (cardiac left-side input, Video 12). For the same imaging plane, a pre-injection B-mode image (left part of Video 13) and color Doppler image acquired with advanced dynamic flow mode (right part of Video 13) are provided. The region enclosed by the dashed line and the two arrows running along both sides indicate the two blood-flow channels within the outflow tract (OFT) separated by the spiral valve. Another arrow indicates blood flow from the outlet on the anatomical right side of the ventricular (V) base, which appears on the superficial side in this right lateral chest-wall approach, into the outflow tract. In this animal, a second injection (pulmonary venous) was performed after confirming adequate washout following the systemic venous injection. In the systemic venous injection (Video 11), it was evident that once more than five cardiac cycles had elapsed after microbubble appearance, recirculating microbubbles became superimposed on the signal.

**Supplemental Video 14. Representative echocardiographic imaging at the spiral-valved outflow region using a long-axis approach from the cardiac apex.**

We found that the long-axis approach from the cardiac apex, as shown in this video, most clearly visualized the spiral-valved outflow region, where blood emerging from the ventricular outlet located on the anatomical right side of the ventricular base, as indicated by the green arrow, flowed through the two channels of the outflow tract separated by the spiral valve (left: B-mode image; right: color Doppler image acquired using advanced dynamic flow mode). L and R indicate the approximate image orientation. V, ventricle; CA, conus arteriosus; TA, truncus arteriosus; SV, spiral valve. However, because the target outflow region was located in the deeper imaging field with this approach, CEUS imaging was not successful owing to strong acoustic shadowing produced by microbubbles flowing through the atrium and ventricle, which were located on the proximal/superficial side. We therefore performed CEUS analysis using a long-axis approach from the right lateral chest wall, which allowed the outflow region to be visualized on the proximal/superficial side (Supplemental Videos 11–13).
